# Addition of MPS-II to the Recommended Uniform Screening Panel in the United States

**DOI:** 10.3390/ijns8040055

**Published:** 2022-10-11

**Authors:** David S. Millington, Can Ficicioglu

**Affiliations:** 1 Department of Pediatrics, Division of Medical Genetics, Duke University Medical Center, Durham, NC 27709, USA; 2 Section of Biochemical Genetics, Division of Genetics, Children’s Hospital of Philadelphia, Philadelphia, PA 19104, USA; 3 Department of Pediatrics, Perelman School of Medicine, University of Pennsylvania, Philadelphia, PA 19104, USA

It has recently been announced that the Secretary of the U.S. Department of Health and Human Services has approved the recommendation by the Advisory Committee on Heritable Disorders in Newborns and Children (ACHDNC) to add mucopolysaccharidosis type II (MPS-II, Hunter Syndrome) to the recommended uniform screening panel (RUSP) in the United States [1]. The implication of this decision is that states that have pledged to abide by the recommendations of the ACHDNC (most of them, in fact—although they are not legally bound to do so) will move towards implementation of newborn screening (NBS) for MPS-II as their budgets and facilities allow. This could take some time—approximately half of the States have still not implemented NBS for Pompe Disease (Glycogen Storage Disease type II, Acid Maltase Deficiency), approved in 2015, and mucopolysaccharidosis type I (MPS-I, Hurler Syndrome), approved in 2016 [2]. Two states—Illinois and Missouri—influenced by special interest groups, have already implemented NBS for MPS-II, and a Taiwanese program started screening for MPS-II in 2015. The published results from these programs [3,4,5] have helped to provide valuable evidence to the ACHDNC to support the nomination. MPS-II is a progressive lysosomal storage disorder characterized by accumulation of the glycosaminoglycans heparan sulfate and dermatan sulfate in lysosomes due to deficiency of the enzyme iduronate-2-sulfatase (I2S), encoded by a gene linked to the X-chromosome, therefore affecting mainly boys.

For those unfamiliar with the process, proposals for adding a new condition to the RUSP may be submitted on-line [6] by parent advocates, organizations or professional experts. A sub-committee conducts a preliminary review of the proposal and presents a summary to the ACHDNC, which decides whether to request a review of evidence by external experts. The full committee is convened after reviewing the evidence and votes on whether to approve the application; a majority vote is sufficient. If successful, final approval must still be given by the Secretary. The overall process can take several years. Frequently, applications are denied on the first submission and may be re-submitted with further evidence, as requested by the committee. In the case of MPS-II, the original nomination was submitted by officials of the National MPS Society in December 2020 and passed initial review in February 2021, then final evidence review and approval one year later, in February 2022 [7]. The nominators claim to have spent 3 years accumulating the evidence requested [8]. The addition of MPS has increased the number of “core conditions” (the primary targets of NBS in the US) on the RUSP to 36 [9]. 

According to the RUSP, a core condition is one that satisfies certain criteria: (i) there is a specific and sensitive test available to detect it; (ii) the health outcomes of the condition are well understood; (iii) there is an available and effective treatment; and (iv) identification of the condition could affect the future reproductive decisions of the family [9]. This may sound straightforward enough, but even after successful recommendations from the ACHDNC, expert opinion varies on whether criteria (ii) and (iii) have been justifiably satisfied, especially in the case of lysosomal storage disorders [10]. 

The initial screening test for MPS-II is an enzymatic assay that determines the residual activity of I2S in dried blood spot specimens. Two methods are available, one based on tandem mass spectrometry [3,5] and the other based on microfluorometry [4]. They appear to be equally effective in detecting newborns at risk [4]. Because there are numerous reasons for low enzymatic activity, it is recommended that second-tier molecular testing and/or biochemical testing [3,4,5] be applied before results are released to mitigate the over-reporting of false positives. Nevertheless, even in newborns with a “confirmed” or “likely” diagnosis based on genetic testing, it is not always clear whether or when to begin treatment because patients, including siblings, sharing the same pathogenic mutations do not always express the clinical symptoms of the disorder at the same age or to the same degree of severity.

The decision of when to treat is challenging because the treatment is both invasive and very expensive [10,11,12]. The current treatment of choice for MPS-II is enzyme replacement therapy (ERT), usually administered weekly via intravenous infusion over a period of hours. Although well-tolerated and known to alleviate many of the clinical symptoms of MPS-II, it is not curative and does not prevent the later development of skeletal abnormalities, nor does it have any impact on the brain, where damage that may have occurred in utero may be irreversible [11,13]. Hematopoietic stem cell transplant (HSCT) therapy, which unlike ERT is potentially able to treat the CNS manifestations of MPS-II, is an alternative treatment that may be considered if ERT is either not available or not recommended. HSCT, however, has limited impact on the somatic symptoms and has other disadvantages that may preclude its application [5,14]. In neonates where there is no apparent brain damage, intrathecal or intracerebroventricular ERT or HSCT may be considered but must be administered concomitantly with ERT [12]. There is no proof that these therapies are effective in preventing damage to the brain in the long-term. On the other hand, MPS-II is a rare condition thought to affect only about 1 in 100,000–150,000 newborns in the USA, which implies that aggressive treatment would be necessary for relatively few newborns. 

Although the current therapeutic strategies for LSDs including MSP-II may be only partially effective, there is hope that more effective options will become available in the future. One approach that has shown some promise is a form of ERT where the enzyme is fused to the C-terminus of the heavy chain of an anti-human transferrin receptor antibody and is thus capable of crossing the blood–brain barrier [14,15]. Gene therapy is also a potential future option—there is a clinical trial of single-dose gene therapy for MPS-II already underway, although it will be several years before their results will be known [16]. In the meantime, wider adoption of NBS for MPS-II remains controversial.
